# Development of an improved construct for spectinomycin selection in plant transformation

**DOI:** 10.1186/s13104-025-07178-3

**Published:** 2025-03-22

**Authors:** Kent F. McCue, Min Shao, Jennie Huynh, Tai Minh, Mandy Chan, York Moy, James G. Thomson

**Affiliations:** 1 USDA-ARS Crop Improvement and Genetics Research Unity, 800 Buchanan Street, Albany, CA 94710 USA; 2 Alpine Bio, South San Francisco, CA 94080 USA

**Keywords:** Transgenic plants, Selectable marker, Spectinomycin resistance, Kanamycin resistance, Gene stacking

## Abstract

Efficient selection of transgenic plants is essential in plant biotechnology, and development of effective selectable marker systems plays a crucial role, especially as multiple rounds of transformation and gene stacking may require multiple selectable markers. The present study aims to develop and evaluate an enhanced spectinomycin resistance construct to provide robust selection for transformation events. The new construct was compared in multiple plant species to the standard spectinomycin selectable marker gene currently used for soybean transformation and to the neomycin phosphotransferase (*nptII*) gene that confers kanamycin resistance. In *Arabidopsis thaliana*, potato, and citrus, the transformation efficiency provided by the enhanced spectinomycin marker was approximately equal to that of kanamycin and considerably better than that observed for the standard spectinomycin marker. In soybean, the enhanced spectinomycin resistance marker preformed as well as the standard spectinomycin gene. These results demonstrate that the enhanced spectinomycin construct provides strong resistance, comparable to kanamycin in multiple species, offering an alternative tool for transgenic plant selection.

**Supplementary Information:**

The online version contains supplementary material available at 10.1186/s13104-025-07178-3.

## Introduction

The development of transgenic plants is a cornerstone of plant biotechnology, allowing for the introduction of new traits such as pest resistance, improved nutritional content, and enhanced stress tolerance. A critical step in this process is efficient selection of transformed plants, which is facilitated by use of selectable marker genes. These genes confer resistance to specific antibiotics or herbicides, enabling the identification and propagation of transgenic cells while inhibiting cell division of non-transformed cells.

Our lab is developing systems to target transgenes to established recognition sites in the genome. Using recombinase mediated cassette exchange (RMCE) technology, we seek to stack genes at a specific locus, while replacing the previous step’s selectable marker gene, thus minimizing the genetic footprint from multiple rounds of transformation [1]. While kanamycin is a robust selective antibiotic for many plant species, our system requires a second reliable selectable marker. Spectinomycin resistance has been used as a selectable marker in chloroplast transformation due to its effectiveness in inhibiting chloroplast protein synthesis. Currently, the best spectinomycin selection cassette consists of a double 35 S promoter and a *Nicotiana tabacum* (Nta) Stromal Targeting Domain (STD) peptide fused upstream of the plant-optimized aminoglycoside-3’-adenylyltransferase A (*aadA*) coding sequence, which is followed by a 35 S terminator ((Nta STD-aadA; Supplemental Figure S1). The Nta STD-aadA construct has been successfully used for soybean transformation for years [2–4]. While use of the *aadA* gene without a Stroma-Targeting Domain yields very low efficiencies during nuclear transformation [5].

In this study we modify the spectinomycin selection gene (STD-aadA) for improved transformation efficiency in multiple plant species. This enhancement was accomplished by fusing the sulfadiazine resistance ORF (*sul1*) with a truncated stroma targeting domain peptide from the pea Rubisco rbcS to the N-terminus of the *aadA* marker gene (Supplemental Figure S2). The *sul1* gene encodes dihydropteroate synthase (DHPS), an enzyme involved in the biosynthetic pathway of folic acid (vitamin B9). Folic acid is required for biosynthesis of purine nucleotides and metabolism of the amino acids serine, glycine, histidine, and methionine. Further, the *sul1* has previously been used for the production of engineered plants without report of deleterious effects [6–7]. The fusion gene cassette (pea STD trunc-sul1-aadA) was evaluated in *Arabidopsis thaliana*, *Carrizo citrange trifoliate* (citrus rootstock), *Solanum tuberosum* (potato), and *Glycine max* (soybean) for transformation efficiency as compared to the standard spectinomycin (Nta STD-aadA) and kanamycin (*nptII*) selection marker genes.

## Results

### Comparative transformation efficiency in Arabidopsis

In *Arabidopsis thaliana*, the transformation efficiency improved from 0.29% with Nta STD-aadA to 1.83% with the pea STD trunc-sul1-aadA construct (Supplemental Table S1). When selecting for the internal *nptII* gene with kanamycin, the transformation event ranged from 1.55% for the Nta STD-aadA to 2.05% for the pea STD trunc-sul1-aadA construct. The sixfold increase between the two spectinomycin selection systems highlights the enhanced functionality of the pea STD trunc-sul1-aadA construct in this model plant. Seeds were prepared independently for each marker experiment using the EHA105 agrobacterial strain. Values are the total of two independent experiments.

**Table 1:** Comparative transformation efficiency in citrus

Marker	Selection	Explants	Total DsRed Shoots	Transformation Efficiency	
pea STD trunc-sul1-aadA	Spec	96	36	37.5 ± 6.3%	
nptII	Kan	96	30	31.1 ± 3.1%	

In citrus, pea STD trunc-sul1-aadA achieved a transformation efficiency of 37.5± 6.3%, compared to 31.1 ± 3.1% with kanamycin (Table 1; Supplemental Table S2). This result indicates that spectinomycin selection using the pea STD trunc-sul1-aadA construct is as effective for citrus transformation as the traditionally used kanamycin strategy. Attempts to use the Nta STD-aadA construct for citrus transformation did not yield transgenic DsRed expressing shoots when using spectinomycin at 25 mg/L. However, when the selection pressure was reduced to 10 mg/L shoots were observed, but most of the plantlets were mosaic or escapes (unpublished observations).

**Table 2:** Comparative transformation efficiency in potato

Marker	Selection	Explants	Putative Events	PCR positive	Transformation Efficiency
Nta STD-aadA	Spec	39	15	12	30.8%
Nta STD-aadA	Kan	40	25	23	57.5%
pea STD trunc-sul1-aadA	Spec	45	36	30	66.6%
pea STD trunc-sul1-aadA	Kan	36	24	18	50.0%

In potato, pea STD trunc-sul1-aadA resulted in a transformation efficiency of 66.6%, significantly higher than the 30.8% efficiency observed with Nta STD-aadA and comparable to the 50.0-57.5% efficiency achieved with kanamycin (Table 2; Supplemental Table S3). Experiments were performed twice; results were totaled but no standard deviation provided.

**Table 3:** Comparative transformation efficiency in soybean

Marker	Selection	Explants	Putative Events	DsRed + shoots	Transformation Efficiency	
Nta STD-aadA	Spec	621	273	63	10.0 ± 4.8%	
pea STD trunc-sul1-aadA	Spec	2114	635	262	12.3 ± 2.4%	

In soybean, the pea STD trunc-sul1-aadA construct was statistically comparable to the traditionally used Nta STD-aadA selection marker, with transformation efficiencies of 12.3 ± 2.4% and 10.0 ± 4.8%, respectively. pea STD trunc-sul1-aadA results are the average of 8 independent experiments, and Nta STD-aadA values are the average of 4 independent experiments (Table 3; Supplemental Table S4).

## Methods

### Plant transformation and regeneration

Two spectinomycin selective gene cassettes were produced. The first cassette consists of the Nicotiana tabacum Rubisco small subunit gene rbcS derived Stromal Targeting Domain signal peptide (Nta-STD) fused upstream of the soybean-optimized aminoglycoside-3′-adenylyltransferase A (*aadA* OPT). The coding sequence, which confers resistance to spectinomycin was, (Nta STD-aadA; Supplemental Figure S1). The second cassette consists of truncated pea (*Pisum sativum*) Rubisco small subunit gene rbcS derived Stromal Targeting Domain signal peptide (pea-STD trunc) fused upstream of a sulfadiazine resistance (*sul1* OPT) ORF with a which in turn is fused to the N-terminus of the *aadA* OPT gene. Both coding sequences were soybean codon optimized, (pea STD trunc-sul1-aadA; Supplemental Figure S2). The Nta STD-aadA and pea STD trunc-sul1-aadA constructs were expressed by the double 35 S promoter / 35 S terminator cassette and inserted into the pCTAGV-KCN3 binary vector [8]. The pCTAGV-KCN3 vector contains the *nptII* resistance and DSRed visible marker genes for identification and comparison of transgenic events. These binary vectors termed pCTAGV-Nta STD-aadA and pCTAGV-pea STD trunc-sul1-aadA (Supplemental Figure S3 & S4), were introduced into the EHA105 agrobacterium strain and used for plant transformation. *Arabidopsis thaliana* was transformed via the floral dip method as per [9]; *Carrizo citrange trifoliate* (citrus) was transformed as described by [8]; *Solanum tuberosum* (potato) explants were transformed according to [10]; and *Glycine max* (soybean) explants were transformed by the method of [11]. Following co-cultivation, the explants were transferred to media containing spectinomycin (25 mg/L) or kanamycin (50–70 mg/L) for generation of callus and shoots. Regenerated shoots were transferred to rooting media, still under selection, to ensure the survival of only transgenic plants.

### Analysis of transgenic plants

*Arabidopsis* seeds were weighed and plated in the presence of either spectinomycin or kanamycin and scored for growth and bleaching. Explants of the other species were grown for production of callus, shoots, and roots in the presence of the selection agent. Transgenic shoots and plants were analyzed visually for the presence of red fluorescence or by PCR for the spectinomycin gene.

### Efficiency of transformation

To evaluate the relative performance of the pea STD trunc-sul1-aadA cassette to the Nta STD-aadA or the *nptII* cassette, the efficiency of transformation for each was calculated as the percentage of explants that successfully produced transgenic plantlets.

## Discussion

The spectinomycin gene with a Stromal Targeting Domain (STD) peptide has been effectively used for soybean transformation, with several patents filed for its used [2–4]. Further, it is an efficient selection marker for chloroplast transformation [12], but it has rarely been employed for production of nuclear transgenic plants other than soybean [13].

Initial attempts at using the Nta STD-aadA gene for spectinomycin based selection in plants other than soybean led to low transformation efficiencies, mosaics and false positive plants due to the low (5–10 ug/L) selective pressure that could be tolerated (unpublished observations). In an attempt to enhance the spectinomycin resistance, the *sul1* coding sequence was fused to the *aadA* gene, both of which were soybean codon optimized. The *sul1* gene encodes dihydropteroate synthase (DHPS), an enzyme involved in biosynthetic pathway of folic acid required for biosynthesis of purine nucleotides and amino acids; moreover, folate metabolism is differentially regulated in response to various abiotic stresses [14]. Further, studies have shown that folic acid biosynthesis is upregulated in actively dividing tissues [15], suspension cells culture [16] and developing embryos [17]. Thus, our lab postulated that the addition of the *sul1* OPT coding sequence would enhance biotic stress tolerance and encourage cell division in the plant during the selective pressure of spectinomycin detoxification.

The effectiveness of the pea STD trunc-sul1-aadA cassette was observed for successful transformation of citrus, potato, and Arabidopsis using spectinomycin selection at 25 mg/L (Tables 1 and 2; Supplemental Table S1-S3). Regeneration of transgenic plants using the plant codon optimized spectinomycin CDS (aadA-OPT) as a selectable agent has not been previously reported in Arabidopsis, citrus, or potato. Our results with the traditional Nta STD-aadA cassette in these species were unsatisfactory, as putative transgenic plants appeared weak and unhealthy under selection pressure (unpublished observations). The present study demonstrates that the pea STD trunc-sul1-aadA was superior to the standard Nta STD-aadA cassette and equivalent to *nptII* selection in these species. As kanamycin is the gold standard for transformation selection, the fact that the pea STD trunc-sul1-aadA cassette was equal to *nptII* highlights its robustness in multiple dicot species. In soybean, spectinomycin has been a reliable selectable marker for transformation for many years [2–4]. The novel design of the pea STD trunc-sul1-aadA cassette may circumvent the numerous patents on the use of Nta STD-aadA cassette, opening its availability for widespread use.

## Conclusion

The results from this study demonstrate significant improvements in transformation efficiency with the codon optimized pea STD trunc-sul1-aadA construct, which not only surpasses the performance of aadA-OPT (Nta STD-aadA) in Arabidopsis, citrus, or potato, but also competes effectively with kanamycin. The pea STD trunc-sul1-aadA construct provides a robust and reliable method for spectinomycin selection of transformed plants, with broad implications for enhancing the efficiency and effectiveness of plant genetic engineering and its use for RMCE facilitated selectable marker swapping required for gene stacking events via sequential transgenic transformations.

### Limitations

Note that spectinomycin selection requires more time for shoots to emerge and develop than kanamycin selection does. The authors do not attempt to determine the exact manner in which of the *sul1* gene assists the spectinomycin selection process.

## Electronic supplementary material

Below is the link to the electronic supplementary material.

**Supplementary information:** Supplementary Figure S1. Schematic and sequence of db35Sp-STD-aadA-OPT-35St (Nta STD-aadA) cassette; Supplementary Figure S2. Schematic and sequence of db35Sp-sul1:aadA OPT-35St (pea STD trunc-sul1-aadA) cassette; Supplementary Figure S3. Schematic of binary vector pCTAGV- Nta STD-aadA; Supplementary Figure S4. Schematic of binary vector pCTAGV- pea STD trunc-sul1-aadA. Supplementary Table S1. Arabidopsis Transformation Data; Supplementary Table S2. Citrus Transformation Data; Supplementary Table S3. Potato Transformation Data; Supplementary Table S4. Soybean Transformation Data

## Data Availability

Available upon request. See supplemental tables for cassette sequences and experimental data.

## Abbreviations

CDS: CoDing Sequence
STD: Stromal Targeting Domain signal peptide
aadA: Aminoglycoside-3’-adenylyltransferase A
OPT: Optimized for soybean protein expression
sul1: Sulfadiazine resistance gene
sul1 OPT: Soybean-optimized sulfadiazine resistance gene
nptII: Kanamycin resistance gene
pea STD-trunc-sul1-aadA: Sul1:aadA cassette
aadA OPT: Soybean-optimized aminoglycoside-3′-adenylyltransferase A
Nta-STD: Nicotiana tabacum Rubisco small subunit gene rbcS derived Stromal Targeting Domain signal peptide
RMCE: Recombinase Mediated Cassette Exchange
